# 1234. Analysis of an Automated Letter HCV Screening Program within a Veterans Affairs Health System: Implications for Universal HCV Screening

**DOI:** 10.1093/ofid/ofac492.1066

**Published:** 2022-12-15

**Authors:** Lucas Harjono, Yash Motwani, Neaka Mohtashemi, Phillip Chen, Greta Tamkus, Tien Dong, Jihane Benhammou, Matthew B Goetz, Jenna Kawamoto, Arpan Patel, Debika Bhattacharya

**Affiliations:** Greater Los Angeles VA, Los Angeles, California; UCLA Health, Los Angeles, California; UCLA and Greater Los Angeles VA, Los Angeles, California; UCLA, LOS ANGELES, California; UCLA, LOS ANGELES, California; University of California, Los Angeles, Los Angeles, California; University of California, Los Angeles and Greater Los Angeles Veteran Affairs, Los Angeles, California; VA Greater Los Angeles Healthcare System, Los Angeles, California; VA Greater Los Angeles Healthcare System, Los Angeles, California; University of California, Los Angeles and VA Greater Los Angeles Healthcare System, Los Angeles, California; University of California, Los Angeles, Greater Los Angeles Veteran Affairs, Los Angeles, CA

## Abstract

**Background:**

Although universal Hepatitis C Virus (HCV) Screening in the US was recommended in 2020, the optimal implementation method is unknown. We characterized the efficacy of an automated letter HCV screening program at the Veteran’s Affairs (VA) Greater Los Angeles Healthcare System (VAGLAHS) and evaluated associations with linkage to care.

**Methods:**

From January 2017 to May 2020, 14,804 Veterans born between 1945-1965 who did not have an HCV antibody (Ab) test result within the last 10 years and who were within the VAGLAHS catchment area were identified. Veterans were mailed a letter recommending HCV screening via a centralized process. Veterans then used the letter to present to a VA laboratory for HCV Ab testing, which included reflex HCV viral load. Those who were HCV viremic were referred to Hepatology/Infectious Diseases clinics for initiation of HCV treatment. Baseline characteristics of those with subsequent HCV viremia were collected. To determine associations with the first HCV visit (linkage to care), we performed independent chi-squared tests.

Flowchart of Automated Letter HCV Screening for Veterans

**Figure:**
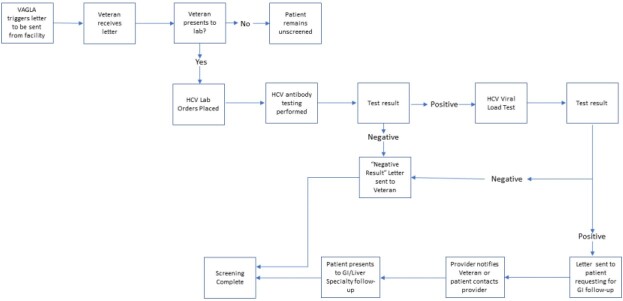

**Results:**

A total of 12,875 Veterans were identified, 4,011 (31%) Veterans presented for HCV Ab testing, 167/4011 were HCV Ab + (4.2%), and 69/167 (41.3%) had HCV viremia. Of those viremic, 94 % were male, 26% were African-American, 62% had stable housing, 24% lived >90 miles from the nearest VA clinic, and 17% had cirrhosis. Fifty-five Veterans (80%) were evaluated in a viral hepatitis clinic and 84 % (46/55) initiated HCV treatment (Figure 1). Patients’ housing status (p = 0.02), cirrhosis (p< 0.0001), and distance to clinic (p=0.063) were associated with non-linkage to an initial HCV appointment.

**Conclusion:**

One third of Veterans approached via mail participated in HCV Ab testing. Overall HCV Ab positivity rates were 4% and nearly half had HCV viremia. The majority of Veterans were linked to care but housing status, cirrhosis, and distance to clinic were associated with non-linkage to care. Automated letter screening is a promising approach to HCV screening, including universal screening. Future research should include investigations of telehealth and e-consults for linkage to care, especially for those who have marginalized housing status and live far from clinic.

**Disclosures:**

**Debika Bhattacharya, MD, MSc**, Gilead: Grant/Research Support|Regeneron: Grant/Research Support.

